# Incidence of Hyponatremia and Associated Factors in Preterm Infants in Saudi Arabia

**DOI:** 10.7759/cureus.23869

**Published:** 2022-04-06

**Authors:** Maha Bamehrez

**Affiliations:** 1 Neonatology, King Abdulaziz University Faculty of Medicine, Jeddah, SAU

**Keywords:** neonatology, nicu, serum sodium, preterm, hyponatremia

## Abstract

Objective: To investigate the incidence of hyponatremia in preterm infants. The secondary aim was to determine the risk factors of late-onset of hyponatremia (LOH) and their influence on neonatal outcomes.

Study design: The present study is a retrospective cohort study of 71 preterm infants born before 32 weeks of gestation at King Abdulaziz University Hospital and admitted to the neonatal intensive care unit, level 3, from January to December 2019. Hyponatremia was defined as a sodium level ≤ 132 mEq/L. Retrieved maternal characteristics included age, parity, booking status, medical problems, antenatal steroids, and method of delivery. Retrieved infant characteristics included birth weight, gestational age, Apgar scores, total parenteral nutrition (TPN), breast milk or formula feeding, and body weight. as well as data on early onset of sepsis, chronic lung disease (CLD), patent ductus arteriosus, retinopathy of prematurity, severe intraventricular hemorrhage, and prescribed antibiotics and diuretics.

Results: Out of 72 preterm infants, 27% were diagnosed with hyponatremia. Early-onset hyponatremia (EOH) affected 7% (n=5) of the infants while 22.5% (n=16) of preterm neonates developed LOH (two infants developed both EOH and LOH). None of the investigated maternal characteristics were significantly associated with hyponatremia. Some infant characteristics were significantly associated with LOH: lower birth weight, lower gestational age, exclusive breastfeeding, longer duration TPN, and use of diuretics and antibiotics. Infants with LOH had longer hospital stays (p < 0.001) and higher risks of extra-uterine growth retardation (p = 0.007), CLD (p < 0.001), and sepsis (p = 0.001).

Conclusion: Twenty-seven percent of preterm infants in King Abdul Aziz University Hospital are born with or develop hyponatremia. Risk factors significantly associated with LOH included lower gestational age, lower birth weight, exclusive breastfeeding, long-term TPN, and prescribed diuretics and antibiotics. Possible outcomes of hyponatremia were long-term hospital stays, growth retardation, sepsis, and CLD.

## Introduction

Fluid and electrolyte imbalances are common in neonates, especially preterm infants. In the first weeks of life, the capacity of the newborn kidney to excrete excess water and sodium is limited, which could lead to respiratory distress syndrome, sepsis, and necrotizing enterocolitis. Since sodium is the primary electrolyte in extracellular fluid, studies on hyponatremia have used varying levels of sodium. Among them, Kim et al. defined hyponatremia as an electrolyte imbalance disorder in infants that occurs when the plasma sodium level is below 132 mmol/L [1].

Furthermore, cohort studies have reported electrolyte imbalance prevalence in preterm newborns of 25%-65% [2-6]. Chowdhury et al. found that about 14.0% of preterm infants born before 32 weeks of gestation had hyponatremia [4]. Al-Dahhan et al. reported that 70% of preterm newborns born at 30-32 weeks of gestation had hyponatremia [5], while Hao found that 29.4% of the preterm infants born before 36 weeks of gestation suffered from hyponatremia [6]. 

Recent studies have shown that one-third of preterm infants suffered from hyponatremia, exceptionally low birth weight [3,7,8]. Another study found that more than a third of very-low-birth-weight infants develop hyponatremia after the first week of life [3]. Nevertheless, the literature has agreed to define hyponatremia that develops in the first week of infant life as early-onset of hyponatremia (EOH), and hyponatremia that develops after the first week of life as late-onset hyponatremia (LOH) [3].

Suggested risk factors associated with hyponatremia in preterm infants include lower gestational age at birth, presence of respiratory distress syndrome, use of diuretics such as furosemide, and feeding with breast milk [9]. Consequently, cerebral edemas and insufficient lung activity are due to symptomatic hyponatremia in infants [10]. Other studies found hyponatremia to be associated with impaired neonatal growth and impaired neurodevelopment at 10-13 years of age [11, 12]. Hyponatremia can further increase the risk of sensorineural hearing loss and cerebral palsy in childhood [13]. Few studies have addressed the incidence of and risk factors for hyponatremia in preterm newborns. To our knowledge, no study has investigated the prevalence of hyponatremia and potentially associated risk factors in preterm infants under 32 weeks gestational age in Saudi Arabia. We hypothesized that there is a positive relationship between hyponatremia in infants and gestational age, birth weight, and the use of diuretics and antibiotics. Thus, the aim of the present study was to determine the incidence of hyponatremia in preterm infants in single-center Saudi Arabia. A secondary aim was to investigate possible risk factors associated with LOH in preterm infants.

## Materials and methods

The research ethics committee at the Unit of Biomedical Ethics, King Abdulaziz University, Faculty of Medicine, Jeddah, Saudi Arabia, approved the present retrospective cohort study (approval number 637-20) conducted at King Abdulaziz University Hospital (KAUH) in Jeddah. The study followed the guidelines of the Declaration of Helsinki. 

Participants

The present study includes all inborn preterm infants with a gestational age of fewer than 32 weeks and admitted to the neonatal intensive care unit (NICU), level 3, in KAUH from January to December 2019. Besides outborn preterm infants, exclusion criteria included the presence of chromosomal or multiple congenital anomalies, death or discharge before two weeks of life, and the presence of any surgical problems. 

Study design

KAUH is an academic and tertiary center with about 4,000 annual births. The NICU has level 2 and level 3 neonatal intensive care beds. Hospital policy states that infants born at ≥ 23 weeks gestational age and weighing more than 500 grams should be resuscitated. The present study retrieved maternal and infant characteristics from the hospital's electronic medical records. The standard medical records were requested if any information was missing in the electronic records. The present study collected serum sodium levels, nutrition and monitoring protocols, and infant and maternal characteristics. 

Serum sodium level

In the present study, serum sodium level was determined corresponding to Kim et al. [1]. Accordingly, we divided patient characteristics into two groups: EOH and LOH. In the first week of life, infants with serum sodium levels below 132 mmol/L were classified as EOH, and after the second week of life, LOH.

Nutrition and monitoring protocol

Enteral and parenteral nutrition was initiated according to the approved feeding guidelines of the Saudi neonatal society [14]. On the first day, infants were given total parenteral nutrition (TPN) including 1-2 g/kg/day protein and 0.5-1 g/kg/day lipids; when hemodynamic stability was reached, enteral feeding was begun. The volume of enteral nutrition was increased by 10-15 mL/kg every day. Parenteral nutrition continued until the volume of enteral nutrition reached 50%-75% of total fluid intake. 

When full enteral feed was reached in infants being fed breast milk, human milk fortifier was added to the diet. The volume of enteral feeding was increased until 10-15 g/kg/d in weight gain was reached. This rate requires total fluids of 150-180 mL/kg/d. 

Serum sodium levels were closely monitored to maintain a level of 140 mmol/L. When the sodium level dropped below 132 mmol/L, a correction was calculated and given either parenterally or enterally depending on the feeding condition of the infant. Corrections varied between 4 and 5 mmol/kg/d of sodium chloride. 

Maternal characteristics

The following data on the mothers were collected: age; parity; medical problems such as diabetes mellitus (DM) and hypertensive disorders; antenatal steroid medication; the presence of chorioamnionitis; and method of delivery.

Infant characteristics

The following data on the infants were collected: birth weight; gestational age (based on the last menstrual period or based on pregnancy testing, prenatal ultrasound, or both); Apgar scores calculated at 5 minutes, duration of TPN (in days); and type of feeding (breast milk, human milk fortifier, formula). Extra-uterine growth retardation was recorded when body weight was below the 10th percentile at age of 35 weeks postconceptional age.

Confirmation of early-onset sepsis (within 72 hours of birth) and late-onset sepsis depended on positive blood culture. Other data on short-term morbidities were retrieved including for chronic lung disease (CLD), defined as the need for assisted ventilation or oxygen supplementation at 36 weeks postconceptional age [15]; for patent ductus arteriosus, from the echocardiogram done when the infant developed hemodynamic instability and necessitated medical or surgical treatment; and for severe intraventricular hemorrhage, from the transcranial ultrasound. The preterm infants were first examined between the third and fifth day of life and thereafter as indicated. In the present study, intraventricular hemorrhage grades 3 and above were recorded using Papile grading [16]. Retinopathy of prematurity was classified using the international classification criteria for retinopathy of prematurity [17].

Besides information on periventricular leukomalacia, from the head ultrasound, information on necrotizing enterocolitis requiring medical or surgical treatment, as classified by modified Bell staging criteria, was retrieved [18]. Data were also collected on prescribed antibiotics such as gentamicin and vancomycin; diuretics such as furosemide, hydrochlorothiazide, and spironolactone; and caffeine. 

Statistical analysis

All analyses were performed using STATA (Stata Statistical Software, Release 12; StataCorp LP, College Station, TX) software. The proportions and means for dichotomous and continuous variables, respectively, were calculated to describe baseline maternal and neonatal characteristics. The proportion and 95% confidence interval (CI) were measured to estimate the incidence of hyponatremia, in general, and of EOH and LOH. The chi-square and student t-tests were assessed to determine the maternal and neonatal factors associated with LOH and to estimate the impact of neonatal hyponatremia on the outcome measures. Statistical significance was determined using the 95% CI and a p-value of < 0.05.

## Results

Patient characteristics

The present study recruited 97 subjects; of these, 25 were excluded due to a short hospital stay (<14 days) or lack of enough information. Hence, the study cohort comprised 72 preterm infants. Table 1 presents baseline maternal and neonatal characteristics.

**Table 1:** Baseline data for maternal and neonatal characteristics Abbreviations:  PROM, premature rupture of membranes; TPN, total parenteral nutrition; HMF, human milk fortifiers.

Maternal Characteristics (n= 72)	
mean (SD)	
Age in years	32.0 (5.7)
	n/Total (%)
Antenatal Steroid Use	48/69 (69.6)
Hypertension	16/64 (25.0)
Diabetes	7/63 (11.1)
PROM	11/65 (16.9)
Delivery n/Total (%)	
Spontaneous Vaginal Delivery	57/72 (79.2)
Cesarean section	15/72 (20.8)
Neonatal Characteristics (n=71)	
Gestational age/weeks n/Total (%)	
< 28 weeks	13/72 (18.1)
28 – 30 weeks	32/72 (44.4)
30- 31 weeks	27/72 (37.5)
Sex n/Total (%)	
Male	28/72 (38.9)
Female	44/72 (61.1)
Birth weight/g Mean (SD)	
< 1000 gm	14 (20.3)
1000 – 1500 gm	46 (66.7)
> 1500 gm	9 (13.0)
Multiple gestation n/Total (%)	
	33/72 (45.8)
Apgar score at 5 min. No. /Total (%)	
7 or more	56/68 (82.4)
Less than 7	12/68 (17.7)
Type of feeding n/Total (%)	
Mixed (breast and formula)	35/69 (50.7)
Mixed with HMF	25/69 (36.2)
Exclusive Breast Feeding with HMF	9/69 (13.0)
Time to reach full feed/days Mean (SD)	
	13.5 (7.6)
Diuretic Use n/Total (%)	
	27/72 (37.5)
Antibiotic Use n/Total (%)	
	40/72 (55.6)
TPN duration/days Mean (SD)	
	11.2 (12.1)

Incidence and risk factors

The incidence of neonatal hyponatremia was 26.8% (n= 19; 95%, CI: 17 - 39%), of which 7% (n = 5; 95% CI: 2 - 16%) had EOH and 22.5% (n = 16; 95% CI: 14 - 34%), LOH. 

None of the maternal characteristics were significantly associated with LOH. On the other hand, the neonatal characteristics that were significantly associated with LOH were lower gestational age, lower birth weight, exclusive breastfeeding, long-term TPN, and the use of diuretics and antibiotics (Table 2).

**Table 2:** Maternal and neonatal factors associated with late-onset hyponatremia in preterm infants (n = 71) in a cohort of all preterm infants born at King Abdulaziz University Hospital between January and December 2019 Abbreviations: PROM, premature rupture of membranes; SVC, spontaneous vaginal delivery; CS, cesarean section; TPN, total parenteral nutrition; HMF, human milk fortifiers; EBF, exclusive breast feeding. Note: *Both breast and formula feeding.

Factors		Late-Onset Hyponatremia, n/Total (%)	P-value
Maternal Factors			
Mother Age	≤ 35 years	10/49 (20.4)	0.521
	> 35 years	5/18 (27.8)	
Antenatal Steroid Use	No	3/20 (15.0)	0.284
	Yes	13/48 (27.1)	
Hypertension	No	10/47 (21.3)	0.198
	Yes	6/16 (37.5)	
Diabetes	No	15/55 (27.3)	0.460
	Yes	1/7 (14.3)	
PROM	No	13/53 (24.5)	0.848
	Yes	3/11 (27.3)	
Delivery	SVD	14/56 (25.0)	0.337
	CS	2/15 (13.3)	
Neonatal Factors			
Gestational age (weeks)	< 28	6/12 (50.0)	0.027
	28 – 30	7/32 (21.9)	
	>30	3/27 (11.1)	
Birth weight (gm)	< 1000	7/14 (50.0)	0.017
	1000 – 1500	7/45 (15.6)	
	> 1500	1/9 (11.1)	
Sex	Male	4/27 (14.8)	0.223
	Female	12/44 (27.3)	
Gestation	Single	9/38 (23.7)	0.804
	Multiple	7/33 (21.2)	
APGAR score at 5 min.	7 or more	11/55 (20.0)	0.111
	7 less than	5/12 (41.7)	
Type of feeding	Mixed*	6/34 (17.7)	0.005
	Mixed* + HMF	4/25 (16.0)	
	EBF + HMF	6/9 (66.7)	
Time to reach full feed in days	≤ 7	2/6 (33.3)	0.117
	8 – 14	6/45 (13.3)	
	> 14	6/17 (35.3)	
TPN duration	≤ 7	4/33 (12.1)	0.023
	8 – 14	5/25 (20.0)	
	> 14	6/12 (50.0)	
Diuretic use	No	1/44 (2.3)	< 0.001
	Yes	15/27) 55.6	
Antibiotic use	No	1/32 (3.1)	< 0.001
	Yes	15/39 (38.5)	

**Figure 1:**
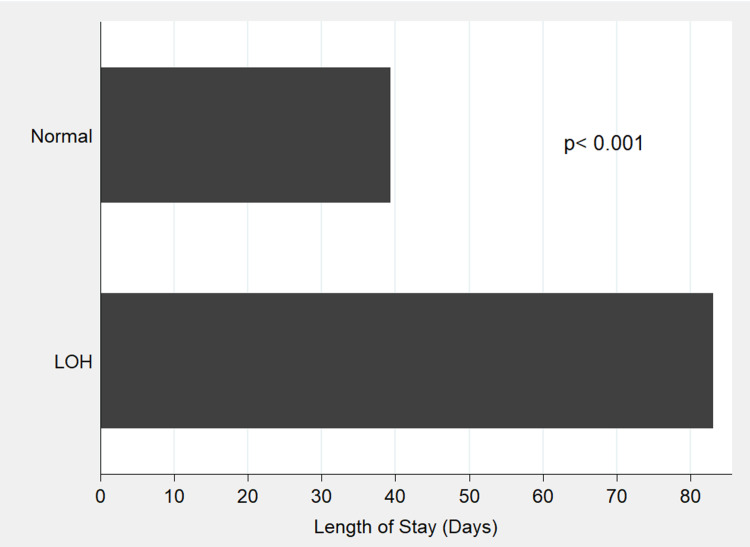
Mean duration of lengths of the hospital stays (in days) of preterm infants with late-onset hyponatremia compared to preterm infant with no hyponatremia Abbreviations: LOH, late-onset hyponatremia.

**Figure 2:**
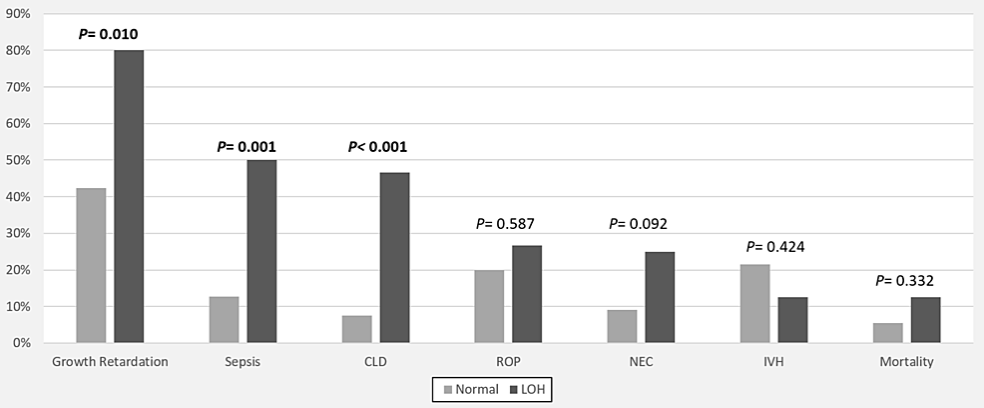
The association between late-onset neonatal hyponatremia and clinical outcomes Abbreviations: CLD, chronic lung disease; ROP, retinopathy of prematurity; NEC, necrotizing enterocolitis; IVH, intraventricular hemorrhage; LOH, late onset hyponatremia.

The LOH was significantly associated with a longer hospital stay (82 vs. 37 days [p < 0.001]) (Figure 1); growth retardation (78% vs. 41% [p = 0.007]); sepsis (47% vs. 12% [p = 0.001]); and CLD (44% vs. 6% [p < 0.001]) (Figure 2).

## Discussion

Sodium concentration in preterm infant serum is the main indicator of the water balance in the body [19]. Small transient variations in the electrolytic balance of the preterm, low-birth-weight neonate, however, are considered normal, in contrast to variations in sodium concentration, for which severe consequences have been recorded. The main aim of the present study was to determine the incidence of and investigate possible risk factors associated with hyponatremia in preterm infants in Saudi Arabia. We found that about one-third of preterm infants born before the gestational age of 32 weeks suffered from hyponatremia. Similar results were reported in Vietnam, where 29.4% of the preterm infants, born before 34 weeks of gestation, suffered from hyponatremia [6], and in South Korea, where 30.4% of preterm neonates, born before a gestational age of 34 weeks, suffered from LOH [1]. In contrast, a recent study carried out in Bangladesh found that fewer preterm infants, born before 37 weeks of gestation, about 14%, suffered from LOH [4].

The present study also found more infants with LOH than with EOH. Likewise, Modi reported that the incidence of hyponatremia increases after the first week of life in low-birth-weight preterm infants [3]. Hence, inadequate sodium intake appears to lead to LOH. Similarly, one study found that inadequate sodium intake and increased natriuresis can lead to vasopressin excretion, which could be another explanation for LOH in preterm infants [9]. The clinical importance of these findings emphasizes the need for early and frequent monitoring of serum sodium levels in preterm neonates to ensure not only appropriate levels of sodium but also to avoid related outcomes. 

Similar to other studies [1,6], the present study found that lower gestational age, low birth weight, and exclusive breastfeeding were factors that were significantly associated with LOH. This finding was expected since breast milk can be an insufficient source of sodium for preterm neonates [20]. Unpredictably, our study findings showed that long-term TPN was significantly associated with LOH. Abnormal antidiuretic hormone sections due to stress and sickness could be one explanation. Of note, one study observed that adult hospitalized patients receiving TPN were more likely to develop hyponatremia than hospitalized patients who were not [21].

The present study also showed that diuretics and antibiotics were significantly associated with LOH. Similarly, other studies have reported that factors, such as the administration of drugs, can exacerbate hyponatremia due to undesirable effects on the kidney tubules [1,6].

One of the many impacts of hyponatremia on preterm infants that our study found was an association between CLD and hyponatremia in premature infants. This finding supports evidence for a strong association between hyponatremia and respiratory distress syndrome in premature infants [1,6]. Studies have suggested that the immaturity of the lungs in a low-birth-weight infant and an electrolyte imbalance can lead to edema and, in turn, insufficient lung activity [10]. Furthermore, neonatal hyponatremia was significantly associated with growth retardation in the present study. Another study found sodium to stimulate cell proliferation and also play a role in protein turnover; thus, it can be considered a significant growth factor [22]. In contrast to our findings, a recent study reported that LOH does not affect the future growth of premature neonates [23].

The association of LOH with the length of the hospital stay is another finding of the present study. Kim et al. found a comparable association [1]. Similar to the findings of a study that found a correlation between electrolyte disturbance and bacteremia [24], our study found an association between LOH and sepsis. A recent study has shown that three-quarters of neonatal sepsis cases suffered from at least one type of electrolyte imbalance [25].

Our study is the first in Saudi Arabia, to our knowledge, that measures the incidence of hyponatremia and possible factors associated with the development of hyponatremia in premature newborns, which can be considered good evidence on which to base future studies in the country. One strength of the present study could be that we included all premature infants during a specific timeframe at the same hospital. Another strength might be that information was collected from both regular patient files and electronic files of the same patients, which can confirm the authenticity of the data. The retrospective nature of the current study and the small sample size, which controls the statistical power to perform multivariate analysis, might be a methodological limitation. A further limitation of this study could be the lack of data on the amount of sodium and fluid intake via TPN: total protein, TG level, and glucose level; this information would have strengthened our study if it had been available. Accordingly, more data need to be included in future studies, such as the amount and content of parenteral fluids being prescribed to the preterm neonate.

## Conclusions

In conclusion, the present study has shown that hyponatremia is not uncommon among premature neonates and, at KAUH, occurs with moderate frequency (27%). Yet, the risk factors that were significantly associated with LOH included lower gestational age, lower birth weight, exclusive breastfeeding, long-term TPN, and the use of diuretics and antibiotics. Our study also found that long-term hospital stays, growth retardation, sepsis, and CLD were possible outcomes of hyponatremia.
